# Why Are Algal Viruses Not Always Successful?

**DOI:** 10.3390/v10090474

**Published:** 2018-09-05

**Authors:** Elena L. Horas, Loukas Theodosiou, Lutz Becks

**Affiliations:** 1 Community Dynamics Group, Max-Planck for Evolutionary Biology, 24306 Plön, Germany; elena.horas@uni-konstanz.de (E.L.H.); theodosiou@evolbio.mpg.de (L.T.); 2 Limnology-Aquatic Ecology and Evolution, Limnological Institute, University of Konstanz, 78464 Konstanz, Germany; 3 Department of Microbial Population Biology, Max-Planck for Evolutionary Biology, 24306 Plön, Germany

**Keywords:** stressors, algal viruses, intrinsic and extrinsic factors, viral life cycle traits, temperature, sunlight, effects, latent period, burst size, host resistance

## Abstract

Algal viruses are considered to be key players in structuring microbial communities and biogeochemical cycles due to their abundance and diversity within aquatic systems. Their high reproduction rates and short generation times make them extremely successful, often with immediate and strong effects for their hosts and thus in biological and abiotic environments. There are, however, conditions that decrease their reproduction rates and make them unsuccessful with no or little immediate effects. Here, we review the factors that lower viral success and divide them into intrinsic—when they are related to the life cycle traits of the virus—and extrinsic factors—when they are external to the virus and related to their environment. Identifying whether and how algal viruses adapt to disadvantageous conditions will allow us to better understand their role in aquatic systems. We propose important research directions such as experimental evolution or the resurrection of extinct viruses to disentangle the conditions that make them unsuccessful and the effects these have on their surroundings.

## 1. Introduction

The invention of the electron microscope around 1930 not only confirmed the existence of viruses but also how diverse they are. The first aquatic virus to be reported was the marine bacteriophage *Photobacterium phosphorium* about 10 years later [1], followed by the isolation of viruses capable of infecting freshwater cyanobacteria [2] and eukaryotic algae [3]. Scientists then began to acknowledge the vast abundance of viruses that were present in aquatic systems, currently recognized to be about 10^6^ to 10^9^ of virus particles per mL of marine waters [4,5] or even higher in freshwater systems [6] and sediments [7]. Viral particles are generally more abundant than any other microorganism, and their abundance is known to be tightly linked to that of the host they infect [8,9]. The recent use of metagenomics has determined that aquatic viruses are not only present in large quantities but that they are also characterized by a very wide genetic diversity worldwide [10,11,12,13,14,15,16,17,18].

Since the discovery of high viral numbers and diversity, their role in aquatic ecosystems has been acknowledged as indispensable in structuring the microbial community and biogeochemical cycles (reviewed, for example, in [6,10,19,20,21,22,23,24,25,26,27]). Briefly, 90% of aqueous systems are made up of microbes which can be roughly divided into two groups: phytoplankton and heterotrophic plankton, both of which are known to be infected by viruses. The structure and diversity of microbial communities can therefore be modified by viruses through selective removal, which can in turn release predation pressure and/or stimulate other trophic levels. Specifically in phytoplankton, viruses are known to be able to remove 25% of the photosynthetically fixed carbon [11] and to restrict phytoplankton blooms [28]. Viruses can also be responsible for the transfer of genetic material between species, through transformation or transduction (to date only reported in bacteriophages), and ultimately for the release of genetic material into the environment after the lysis of the host, making it available to other species. The role of aquatic viruses in biogeochemical cycles—mainly nutrient and energy cycling—involves the movement of material from living organisms into the particulate and dissolved pools of organic matter, referred to as the viral shunt [26]. This is very important as it increases respiration in the oceans and freshwater systems, reduces the direct transfer of microbes to higher trophic levels, increases primary productivity by making more nutrients available for autotrophs, recycles carbon more intensively within the microbial loop, removes carbon from the classical grazing food web by diverting it to the microbial loop and increases carbon that is exported to the deep ocean by the biological pump.

Viruses that infect eukaryotic algae include a wide range of morphologies and genome sizes. Examples include dsDNA viruses such as MpV or PBCV-1 that infect the prasinophyte *Micromonas pusilla* [29] and chlorophyte *Chlorella variabilis* [30], respectively; ssDNA viruses that infect some marine diatoms from the Chaetoceros genus such as CsNIV infecting *Chaetoceros salsugineum* [31] or CdebDNAV infecting *Chaetoceros debilis* [32]; dsRNA such as MpRNAV01B that also infect the prasinophyte *Micromonas pusilla* [33]; and ssRNA viruses such as RsRNAV that infect the diatom *Rhizosolenia setigera* [34] or those that infect some *Heterocapsa* dinoflagellates such as HcRNAV34 infecting *Heterocapsa circularisquama* [35]. The genetic diversity of algal viruses is also recognized to be very large, as shown for example by Cottrel and Suttle [36], who found similar levels of diversity within and across local populations of the viruses that infect the green algae *Micromonas pusilla*. More recently, Van Etten et al. [37] also detected high genetic diversity among the genomes of 43 viruses that infect zoochlorellae algae.

**Figure 1:**
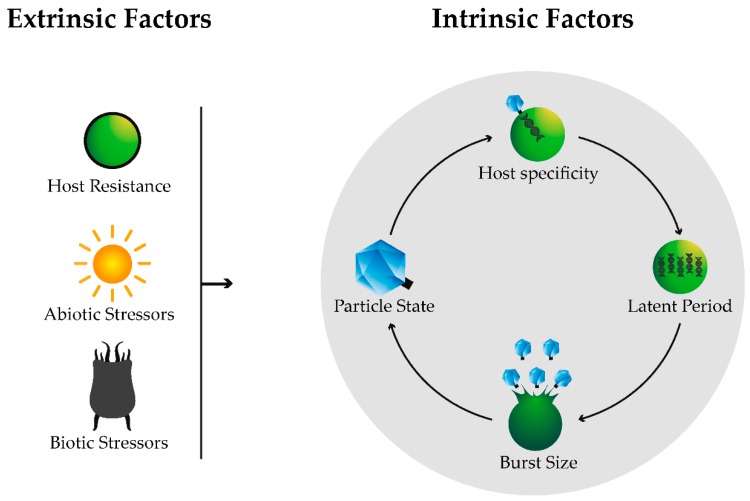
Intrinsic and extrinsic factors that can make viruses unsuccessful during their life cycle stages. Host specificity refers to the attachment and insertion of genetic material (DNA or RNA) into the hosts, latent period refers to the duration of the viral infection within the host during which virus particles are produced, burst size refers to the number of viral progeny released and particle state refers to the state where viruses are not within a host. Extrinsic factors are divided into host resistance mechanisms, abiotic and biotic stressors, and they can affect viruses during any of their life stages.

The effects that algal viruses exert on their environments are of global importance as phytoplankton activity is indispensable in all aquatic systems on Earth. The degree of these effects highly depends on the turnover rate of viruses. If turnover rates are high and rapid, the immediate effects on the aquatic microbial community and biogeochemical cycles will be greater. Therefore, here we aim to elucidate the factors that on the contrary slow down viral turnover rates, consequently also lowering the strong immediate effects that viruses have on their surroundings. These effects will decrease when (i) there are low numbers of viruses in the environment; (ii) they have low or no viral infectivity and/or (iii) they have low or no viral replication ability and subsequent low or no release of viral particles into the environment. Thus, for the purposes of this review, viruses with one or more of these characteristics will be referred to as “unsuccessful”. The factors that make viruses unsuccessful can be either intrinsic to them, when they are associated with their own heritable life cycle traits such as burst size or latent period, or be extrinsic to them and be caused by the environment such as by temperature or host resistance mechanisms (Figure 1). Examples of viruses that infect eukaryotic algae will be reviewed and, as they require host organisms to reproduce, some of their hosts’ physiological traits will also be considered. Finally, we describe why algal viruses might be successful in the long-term despite the many conditions and factors that can lower their success and outline approaches that could be used to study viral adaptations to such conditions.

## 2. Intrinsic Factors

### 2.1. Host Specificity

The first step that determines the success of viral infections is the host–virus encounter. This primarily depends on both host and viral abundances but it is also influenced by other properties such as cell size, swimming abilities or the presence of non-host organisms [20]. Mathematical models based on transport theory have been applied to test how these traits affect host–virus encounter rates. They are assumed to be collision frequencies and to be dependent on the physical properties of the cells and the medium; water in this case [38,39]. Encounters are followed by successful viral attachment and the injection of genetic material into the host cell. Although the mechanism by which algal viruses attach to their hosts has not been exhaustively described to date, it is known that compatibility between host and virus is crucial for successful infection, as tested by Tarutani et al. [40]. Viruses are known to range from extreme specialists to broader generalists [9]. Some viruses are able to infect genetically different host strains from the same species, some are only able to infect one single host strain, some are able to infect hosts from different but closely related species, and some have even been found to infect hosts from distantly related species [20].

In terms of algal viruses, intraspecific infectivity has been observed in several cases such as that of the raphidophyte *Heterosigma akashiwo* and its virus HaV [41], the ssRNA viruses that infect the dinoflagellate *Heterocapsa circularisquama* [35] or those that infect the diatom *Rhizosolenia setigera* [34], the ssDNA viruses that infect the diatoms *Chaetocerus debilis* [32] and *Chaetocerus lorenzianus* [42], or the dsDNA that infects *Phaeocystis pouchetii* [43] among others. On the other hand, interspecific infectivity has not been reported and there are only a few observations pointing at viruses that infect the brown algae *Ectocarpus siliculosus*, from the phaeovirus genus, as also being capable of infecting other algae such as *Kukuckia kylinii* [44,45]. These cross infections of phaeoviruses usually cause deformities in the host but they are not always infectious. This occurs, for example, when EsV-1 virus infects *Feldemannia simplex* or when EfasV virus infects *Ectocarpus siliculosus* [46] and *Myriotricha clavaeformis* [47]. Even though interspecific infectivity has not been extensively reported in algal viruses thus far, there are two main indications that it might be present but has not been discovered yet. First, in aquatic bacterial virology, it has been established that the ability to infect different host species increases when hosts are very diverse but not very abundant [21,48]. Secondly, it has been shown that viruses with around 90% similarities in their genomes differ mainly in the areas that encode for tail proteins which determine host specificity [49]. It is therefore possible that algal viruses in low-productivity areas have evolved the ability to infect hosts from more than one species. This would increase the success of algal viruses, as broader infectivity ranges increase the chances of encountering and successfully infecting suitable hosts.

### 2.2. Life Cycle

Latent period and burst size are the two main life cycle traits that determine whether a virus is successful or not and are commonly specific to every virus. For example, the dsDNA virus PBCV-1 infecting *Chlorella variabilis* has a latent period of 6–8 h and a burst size of around 1000 virus particles with only 30% infectious ones [30], while the ssRNA virus HcRNAV34 that infects *Heterocapsa* circularisquama has a latent period of 24 h and produces 3.4 × 10^3^ infectious particles [35]. It is important to note that the features of these two intrinsic traits can be modified by external cues such as the environment. The focus here, however, is to describe which features of the latent period and burst size make viruses unsuccessful based on our definition of success above.

#### 2.2.1. Latent Period

The latent period is referred to as the duration of the viral infection within the host and can be divided into two types: lytic or lysogenic [50]. While lytic viruses are characterized by causing host cell death and releasing high numbers of progeny quickly after infection, lysogenic viruses are able to form stable interactions with their hosts by integrating their genetic material into the host’s genome or by maintaining it as plasmids (referred to as pseudolysogeny). They do not cause immediate host death and instead are able to remain silent, often for many generations, until a certain condition activates their lytic cycle [6,19,20,23,51]. Some viruses are also able to form chronic infections with their hosts by releasing progeny into the environment through budding and never causing their deaths [52].

Following our definition, lysogenic viruses are less successful than lytic viruses [26]. Their immediate effects on the ecosystem are weaker as they produce a lower number of progeny than lytic viruses in the same period of time. Lytic infections are very quick, and examples within the aquatic realm are fairly common. They include viruses that infect the cocolithophore *Emiliana huxleyi* [53] or *Chlorella*-like algae [30]. On the other hand, lysogenic viruses that infect aquatic algae are to date only found within the phaeovirus group (from the phycodnaviridae family). They include EsV-1, FsV and FirrV-1, which respectively infect *Ectocarpus siliculosus*, *Feldmannia* species and *Feldmannia irregularis*. Their infection strategies are somewhat remarkable as they only infect and integrate their genetic material into the genomes of the algae’s free-living gametes. This ensures that their DNA is mitotically transmitted to all cells during gamete development, although it is only active in the cells of the reproductive organs. Viral replication and release occur when conditions are favourable for gamete release, allowing new viral particles to easily infect newly released gametes [54,55,56].

Despite having grouped this life strategy as one of the ways in which viruses are not very successful, it is also important to note that it represents a survival strategy for them in areas with low host abundance or low productivity and also allows them to be protected in harsh environmental conditions, and it can prevent secondary viral infections in the hosts [28].

#### 2.2.2. Burst Size

Burst size refers to the amount of viral progeny that is released into the environment after infection [57]. Although a larger burst size does not directly indicate a higher number of viable progeny, the chances of releasing infectious viruses increase with an increasing number of progeny. Larger burst sizes have stronger immediate consequences on the environment and are therefore a trait of more successful viruses.

**Figure 2:**
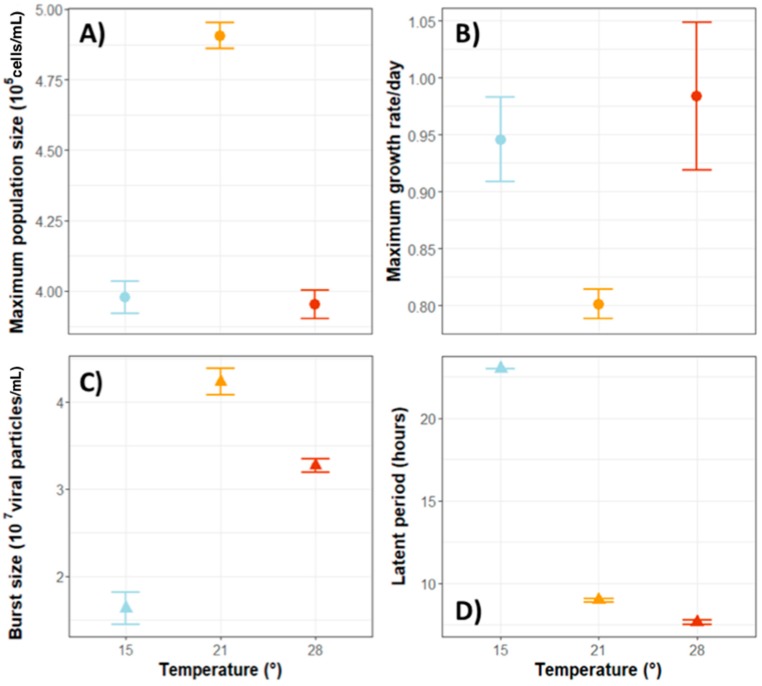
Population dynamics of the algae *Chlorella variabilis* (**A**,**B**) and its virus PBCV-1 (**C**,**D**) when exposed to temperatures of 15 °C (blue circles and triangles), 21 °C (orange circles and triangles) and 28 °C (red circles and triangles). We followed the growth of the algal host *Chlorella variabilis* (strain NC64A) over 21 days at the three temperatures (*n* = 6; modified BBM medium, constant light and shaking as in [87]) and calculated maximum growth rates and maximum population sizes from these data (**A**,**B**). Growth rates were the lowest at 21 °C and population sizes significantly highest (ANOVA: F_2_,_15_ = 18,7, *p* = 8.5 × 10^−5^; posthoc: 15–21 °C: *p* = 0.0003, 15–28 °C: *p* = 0.0002). Virus burst sizes and the length of the latent period (**C**,**D**) were estimated from experiments in which we followed the number of free virus particles post infection (~every hour, for 23 h) at the three temperatures (*n* = 6, initial MOI = 10, modified BBM medium, constant light, and shaking). The number of viral particles were counted using flow cytometry (as in [87]). Virus burst size was significantly smallest (ANOVA: F_2,15_ = 13.516, *p* = 0.0004; posthoc test: 15–21 °C: *p* = 0.0004, 15–28 °C: *p* = 0.014, 21–28 °C: *p* = 0.004) and latent time significantly longest (ANOVA: F_2,15_ = 217.5, *p* < 2.2 × 10^−16^; posthoc test: 15–21 °C: *p* = 0, 15–28°C: *p* = 0, 21–28 °C: *p* = 0.004) at 15 °C. Shown are means and ± standard errors.

Burst sizes are very variable across all known viruses, ranging from hundreds to tens of thousands per cell. Viruses that belong to the phycodnaviridae family, for example, have burst sizes of 10^2^ to 10^3^ viruses per cell [20]. The number of viral particles released into the environment after replication depends highly on intrinsic viral traits but can also vary as a consequence of a host’s traits or environmental cues. Generally, larger host cells and small RNA viruses tend to have larger burst sizes compared to smaller hosts and DNA viruses, respectively [58]. Regarding external conditions, burst size is usually sensitive to the physiological conditions of the host and to environmental factors such as salinity, temperature (Figure 2) or light [57]. These can be separate reasons, but can also be connected, i.e., environmental factors can modify a host’s physiological conditions which can in turn alter the viral burst size.

## 3. Extrinsic Factors

Factors that are not related to the heritable life cycle traits of viruses are grouped here as extrinsic factors. They determine whether a virus is successful or not and are common to the environment. Extrinsic factors can affect viruses during their particle state by increasing viral decay, e.g., low host numbers in the environment decrease the number of successful infections through lower host–virus encounter rates, but they can also modify some of the viruses’ intrinsic life cycle traits, e.g., low temperatures or nutrient-depleted conditions can elongate viral latent periods.

### 3.1. Host Resistance

Host resistance to viral infections is a common factor by which viruses become unsuccessful. To date, different forms of resistance have been reported in microbial hosts such as bacteria or phytoplankton, and they are all related to the different stages of viral infection: attachment, when the virus physically attaches to the algal cell; viral entry, when the virus or its nucleic acids enters the cell; or the last internal steps, which involve viral gene expression, replication, virion assembly and cell lysis [59].

Attachment to the host cell represents the first stage of viral infection and often involves receptors at both ends. Viral receptors include a wide range of proteins, carbohydrates and lipids, allowing them to infect a wide range of hosts. However, hosts have proven to evade this stage by modifying their cell wall receptors, either by decreasing the number of receptors or simply by losing them [59]. Other mechanisms of viral avoidance include the extracellular production of viral inhibitors, such as the cell wall sulfated polysaccharide produced by the red microalga *Porphyridium* [60]. Evans et al. [61] also found that the secretion of cleavage products such as sulfide or acrylic acid has inhibitory effects. These molecules have also been reported to have internal effects by preventing viral protein synthesis or by inhibiting the activity of essential proteins in retrovirus replication [60]. Algal species have also been reported to form colonies with an outer skin, i.e., a biofilm-like structure, that makes them resistant to viral attack [59]. Jacobsen et al. [62] found that the colonial stage of the algae *Phaeocystis pouchetii* could not be infected by the virus PpV-AJ96. This stage consists of *Phaeocystis* cells enclosed by a thin but strong semi-permeable skin with plastic and elastic properties [63]. This skin, also referred to as mucus, is possibly what prevented the penetration of the virus into the cell as the algal free-living form became infected. Other studies, supported by mathematical modelling, indicate that instead it might be a consequence of lower encounter rates between the virus and the colonial algal stage [64]. Using a three-species system (host *Chlorella variabilis* algae, PBCV-1 virus and *Brachionus calyciflorus* rotifer) Frickel et al. [65] found a positive correlation between algal colony formation and their resistance range in response to the virus, but not the rotifer, indicating that resistance evolution might involve growth in colonies. Kimura and Tomaru [66] showed that the diatom *Chaetoceros tenuissimus* was also able to resist viral infection of CtenRNAV during its stationary phase of growth but only in the presence of bacteria, as they observed a bacteria–algae association that inhibited viral infection. Some organisms are also capable of evading viral infection by escaping. The algae *Emiliana huxleyi,* for example, is able to undergo meiosis in the presence of a virus and therefore transform itself from a virus-susceptible diploid cell to a resistant haploid cell. This study also offered Frada et al. [67] the opportunity to introduce the strategy of the “Cheshire cat”, by which cell modification makes organisms invisible to viruses. Studies have also shown that algal life stage can greatly influence their susceptibility to viruses. Some algal species are more susceptible to viruses during the exponential phase of growth than during the stationary phase [68,69,70].

Despite the lack of knowledge regarding mechanisms to block viral entry or replication in marine organisms, some general ones such as the destruction of incoming virus DNA by restriction modification systems or the CRISPR/Cas system have been shown in bacteria [25]. Although there have been no findings of such systems in eukaryotic algae so far, Tomaru et al. [71] were able to show the suppression of viral genome replication in the marine dinoflagellate *Heterocapsa*. By artificially introducing the viral RNA into the cells, they reported that the transfected HcRNAV34 viral genome was replicated only in susceptible cells and not in resistant ones.

Lysogeny, pseudolysogeny or chronic infections can be associated as a host resistance mechanism to prevent or delay host burst and viral release [25]. In an elegant study, Thomas et al. [51] showed that clones from three different genera of Mamiellale green algae (*Bathycoccus* sp., *Micromonas* sp. and *Ostreococcus tauri*) were able to evade lysis after inoculation. They showed that resistance mechanisms in *Ostreococcus tauri* were achieved either through lysogeny or attachment inhibition.

### 3.2. Biotic Stressors

Apart from the ways in which hosts themselves can evade infection by viruses, there are other stressors in the environment that can lower viral success. Here, we review different biotic pressures that directly or indirectly affect viruses.

#### 3.2.1. Predation

Predation represents a direct stressor on viral populations. In the marine realm, nanoflagellates and sponges are known for removing high quantities of viruses through phagotrophy and filter feeding, respectively [72,73]. Because viruses attach to organic particles in the environment, predation of these particles is also involved in viral decay [74]. Preferential grazing of infected cells has also been observed in *Phaeocystis globosa*. A reduction in the abundance of the virus and its progeny occurs due to a secretion of organic compounds by the algae prior to viral lysis [75]. The effects of predation have been reported to account for only 1% of the total viral decay [72]. However, more work needs to focus on the predation on viruses, as virus particles are highly abundant and can provide resources in otherwise resource-limited conditions.

#### 3.2.2. Virophages

Although virophages represent a great threat to other viruses, none that infect phytoplankton cells have been isolated to date. Reported virophages are small, double stranded DNA viruses that require the co-infection of another virus to replicate. They are considered as parasites of larger viruses, specifically some members of the Mimiviridae family, as they take advantage of their replication machinery and often deactivate them or decrease their burst size [76].

#### 3.2.3. Indirect Interactions

The presence of species with which viruses do not directly interact can also affect them. These effects are referred to as indirect and they have been largely studied in ecology [77]. Some examples involving viruses include the exhaustive study carried out by Weinbauer et al. [78]. They proved that several bacterial species were affected differently depending on the type of predator that they were exposed to, either viruses or grazers, and that the number of such predators was also affected. Using a system where the host species was the algae *Chlorella variabilis*, Frickel et al. [65] demonstrated that the evolution of resistance to the virus in the algal host allowed the coexistence of two algal consumers, a rotifer and the virus, which was not possible with only susceptible algal hosts. Without resistance evolution, both consumers competed for the same resource and the virus always outcompeted the rotifer. The presence of the second consumer, however, indirectly slowed down the algal evolutionary changes. Moreover, in a recent review, Sandaa et al. [79] assessed the idea that an alteration in the microbial food web due to predation or the availability of limiting nutrients could strongly affect viral abundances. DeLong et al. [80] showed that predators of *Paramecium bursaria*, a symbiotic ciliate that hosts *Chlorella* algal species, can influence the abundance of chloroviruses. Finally, host resistance mechanisms also have the potential to deconstruct the whole microbial community by making virus-resistant cells resistant to grazing activity as well [81].

### 3.3. Abiotic Stressors

There are many abiotic stressors in the environment that can make viruses unsuccessful. Some stressors can have strong direct effects on viruses when they are outside the host in their particle state through inactivation or destruction, but they can also modify viral life cycle traits by reducing burst sizes or lengthening latent periods. Abiotic stressors include biological, chemical and physical factors. Here, we discuss temperature, sunlight and UV radiation, nutrient concentrations, non-host particles, CO_2_ and salinity.

#### 3.3.1. Temperature

Studies on the effects of temperature on viruses have yielded a variety of results depending on the viral species examined as well as on the time and/or spatial scale of the study. However, the general trend suggests that very high temperatures are detrimental to viral abundance [82].

For example, a dsRNA virus capable of infecting the microalga *Micromonas pusilla* was shown to be inactivated at temperatures between 40 °C and 95 °C [83]. However, temperature effects are so variable that some viral strains from the same species show different effects. Kimura and Tomaru [84] found that several strains of the virus that infects the marine planktonic *Chaetoceros tenuissimus* were negatively affected by a range of different temperatures. Also, strains of the alga *Phaeocystis globosa* showed different sensitivity to temperature changes. While one strain became sensitive at temperatures higher than 35 °C and completely inactive at 45 °C, another strain became sensitive and inactive at 10 °C lower, respectively. Interestingly, they also characterized both strains and found that while the former one was larger in size, with both its genome and proteins being more complex, the latter one had a shorter structure but was also associated with a longer latent period and more specific infections [85]. One of the main effects of temperature is exerted on the structure and elasticity of proteins and membrane lipids, indicating that temperature effects on viruses will mainly depend on viral structures and sensitivities [74]. It is interesting to note that temperature effects can also disrupt host–virus interactions. A study carried out by Friedrichsen et al. [86] showed that the algae *Chlorella variabilis* and its virus PBCV-1 experience contrasting growth rates depending on the temperature they are exposed to. On the one hand, *Chlorella variabilis* population grew slower (Figure 2A) but achieved higher densities (Figure 2B) at intermediate temperatures than at lower or higher ones. On the other hand, the viral population was only affected by low temperatures. At 15 °C, viruses showed smaller burst sizes (Figure 2C) and longer latent periods (Figure 2D).

#### 3.3.2. Sunlight and UV Radiation

Solar and UV radiations are possibly the most important abiotic stressors for algal viruses as their hosts are photosynthetic organisms that rely on light for energy and survival. Algal viruses are therefore in constant exposure to sunlight and UV radiations [88]. The negative consequences of these stressors are linked to protein degradation, structure alteration and eventually a decrease in infectivity. Even though the effects are relatively easy to quantify in laboratory experiments, in natural environments, they depend on several other factors such as the amount of real damaging radiation, the optical clarity of the water or the depth at which the virus is located, making them harder to measure [57]. It is clear, however, that total darkness has negative consequences for algal viruses. Examples include those that infect the algae *Micomonas pusilla*, as their infections are lowered or even prevented in darkness [29], the *Chlorella* virus PBCV-1, whose burst size is reduced by around 50% in the dark [30], or the virus PpV01 that infects *Phaeocystis pouchetii*, which experiences a reduction in burst size from 370 to 100 without light [89]. Moreover, Thyrhaug et al. [90] found that viruses that infect *Pyramimonas orientalis* algal cells produced considerably more progeny when they infected hosts at the end of the dark period (night time) than when they infected hosts during the light period (day time) or at the beginning of the dark period. On the other hand, studies on DNA and RNA viruses that infect the raphidophyte *Heterosigma akashiwo* showed that their latent periods were of similar duration in darkness as in the light [91,92].

Jacquet and Bratbak [93] looked at the effects of UV radiation on the viruses EhV, PoV and PpV and found that only UVB had important negative consequences for them. They also showed that the degree of these effects varied and were species-specific. Abundances of EhV, for example, were lowered in the presence of UVB radiation. PpV and PoV did not show decay under the same conditions but PpV showed complete inactivation. A study found that cool white fluorescent illumination increased viral decay of viruses such as HaV which infects the raphidophyte *Heterosigma akashiwo*, HcV and HcRNAV, both of which infect the dinoflagellate *Heterocapsa circularisquama* [94].

The negative effects caused by sunlight and UV radiation are, however, seasonal as light intensity is stronger and lasts longer in summer than in winter. A long-term study of two viruses that infect *Chlorella*-like algae, ATCV-1 and CVM-1, and a virus that infects *Chrysochromulina parva*, CpV-BQ1, showed that decay rates are the highest during summer and the lowest during winter. This might also be explained by seasonal temperature changes [95].

#### 3.3.3. Nutrient Concentrations

Concentrations of inorganic nutrients such as phosphorus (P), nitrogen (N), silicates (Si) or iron (Fe) determine and limit algae growth in all aqueous systems [74]. Because algal viruses depend on their hosts for successful replication, their concentrations tend to be higher at the shore than in the open ocean and to fluctuate between seasons, following the dynamics of the primary production [19]. Algal viruses are therefore strongly influenced by their hosts’ stoichiometry [96]. Numbers of prasinoviruses, for example, have been shown to increase in areas with higher concentrations of phosphates, nitrates, nitrites, ammonium and silicates independently of their locations [97]. Moreover, a study carried out by Clasen and Elser [98] reported that more PBCV-1 viral particles were released when their *Chlorella variabilis* hosts had low C:P ratios (carbon to phosphorus). Other studies that have used the algae *Emiliana huxleyi* and its viruses as model systems have shown that viral production increases in high nutrient concentrations and N-depleted conditions but that it is 70% lower in P-depleted environments [99]. In viruses that infect the marine picoeukaryote *Micromonas pusilla,* P limitation led to a 150% prolongation of the latent period and an 80% reduction in viral burst sizes [100]. Another study that used the same model system showed that in P-replete conditions, viruses increased by almost five-fold in numbers and that 70% of host cells had lysed after infection compared to 30% in P-depleted conditions [101]. The combination of depleted P and N conditions lowered burst size from 130 to 70 particles in the virus PpVO1 that infects *Phaeocystis pouchetii* [102]. Furthermore, Maat and Brussaard [103] showed that P is not the only environmental nutrient that strongly affects algal viruses. They found that the burst size of PgV was more reduced under N-limitation conditions (92%) than under P-limitation (70%). They also showed that P and N depleted conditions lengthened viral latent periods and reduced burst sizes. All these studies demonstrate that viral distribution and abundance are not only explained by host densities. As Finke et al. [104] reported, incorporating nitrogen and phosphorus concentrations into models provides more confident explanation of viral abundance.

#### 3.3.4. Non-Host Particles

The presence of non-host particles in the environment, such as clay sediments or host debris, can have direct or indirect effects on the success of a virus. Due to the peptide structure that allows viruses to attach, their binding affinity is fairly high and they can sometimes adsorb to particles that are not their hosts [105]. These interactions with non-host particles are, however, highly dependent on the type, size or age of the particles [21]. Interestingly, positive effects have also been reported when attachment is reversible as it can protect viruses from other abiotic stressors [74].

There were reports in the 1990s suggesting that phytoplankton bloom formations cause an increase in DOM (dissolved organic matter) which in turn leads to an increased number of bacteria and a high non-host absorption of viruses [57]. Baudoux et al. [106] suggested that transparent exopolymeric particles (TEP) generated when colonies of the algae *Phaeocystis globosa* are disrupted could be a cause of death in viruses. TEP are very sticky particles that exhibit the characteristics of gels and consist predominantly of acidic polysaccharides. They are present in freshwater as well as marine systems and they can be formed abiotically from dissolved matter or through dead phytoplankton. Large quantities of viruses, from 10–80%, were adsorbed to TEP in *Phaeocystis globosa* mesocosms [107]. Mari et al. [108] also conducted a study on this issue and found that viral abundance decreased with TEP size and that the number of viruses attached to TEP was higher in areas with low water movement. Intriguingly, they also concluded that, due to the short distance between viruses and hosts on TEP, these particles may serve as hot spots for viral infection in cases where adsorbing to particles is reversible.

#### 3.3.5. CO_2_ Concentrations/pH

High CO_2_ concentrations and the associated low pH have been linked to higher viral decay in aquatic systems. Larsen et al. [109] as well as Highfield et al. [110] found that abundances of EhV particles and a group of unidentified dsDNA viruses that infect nanoeukaryotes decreased with increasing CO_2_ concentrations. Burst sizes have also been shown to be negatively affected in EhVs and PgVs, being reduced from 2890 to 2032 viruses per host cell [109] and by almost 14% [111], respectively. Lower burst sizes in high CO_2_ concentrations are due to less available energy for viral replication within the host cell [111].

#### 3.3.6. Salinity

Few studies have focused on the effects of salinity on algal viruses. While Guixa-Boixareu et al. [112] found an indirect impact of increased salinity on viruses, no studies have reported on the direct consequences that changes in salinity can have on algal viruses. However, these effects are thought to be variable and highly dependent on viral permeability to ion concentrations, which is determined by their capsids’ morphology [74]. Future studies should look into the effects of changing salinity on algal viruses as warmer temperatures are causing sea levels to rise and salinity to decrease worldwide.

## 4. Virus Evolution

Viruses might be characterized by a rapid evolutionary potential due to their high standing genetic variation, large population sizes, short generation times, high mutation rates and small genome sizes. They are likely able to quickly evolve and adapt to conditions that make them unsuccessful. Host specificity, latent period and burst size are virus traits that might therefore be strongly selected. As viruses depend on their hosts to reproduce, the evolution of these viral traits depends highly on host state and density. For example, viruses have evolved broader specificities and more extended latent periods in areas with low host abundance or shorter latent periods and larger burst sizes when their hosts are fast growing [113].

Host resistance is an important host trait that determines virus reproduction. Host defences make viruses unsuccessful as they lower or inhibit their infectivity abilities. However, experimental evolution studies of host–virus population coevolution have shown that viruses are also able to quickly evolve counter adaptations and overcome host resistance. This in turn has been shown to lead to an arms race by which adaptations in each species triggers new adaptations in the other, and so forth [114]. On much longer time scales, coevolving with their hosts has also allowed viruses to acquire genes from them, as shown in comparative genomic studies (Table 1). These genes have made viruses more successful in their infections as they usually encode for critical steps of the host metabolism such as photosynthesis, nucleotide metabolism, carbon metabolism, phosphate metabolism or stress response. Horizontal gene transfer allows them to mimic some of the host’s actions and increase their reproductive potential [21]. This has mainly been reported in cyanobacteria/phage systems, but Monier et al. [115] provided phylogenetic proof that it has also occurred in a eukaryotic system: the algae *Emiliana huxleyi* and its virus. Regarding extrinsic factors that affect viruses and make them unsuccessful, the most notable adaptations are linked to resistance mechanisms against solar and UV radiations. Many studies have shown that viral replication inside the host cell occurs during the day but that cell lysis and the subsequent release of viruses occurs at night time. Thus, viruses encounter and infect new algal hosts when their major abiotic stressor is not present. Replicating during the day also gives viruses the opportunity to use the alga’s photosynthetic products as energy for replication and assembly [57,74]. Aligning with these predictions are the studies carried out by Thyrhaug et al. [90] (described in Section 3.3.2); Jacquet et al. [116], who proved that viruses of the algae *Emiliana huxleyi* are characterized by a diel pattern with more progeny being produced during dusk; or Derelle et al. [117] who showed diurnal patterns of infection in prasinoviruses. Furthermore, a homolog of the denV gene has been found in chloroviruses. This gene encodes for a repair enzyme involved in pyrimidine photodimer excision and indicates that chloroviruses might be able to recover after high exposure to solar radiation through photoreactivation [118].

## 5. Conclusions and Outlook

Here, we provide a review of the conditions that can make viruses unsuccessful. Understanding these conditions as well as when and how viruses may overcome them will help us elucidate the overall success of aquatic viruses and their role in shaping aquatic communities and biogeochemical cycles. The rapid evolution of viral populations is likely to be a key factor that has allowed aquatic viruses to succeed and diversify. Future work should thus focus on the evolutionary potential of viruses determining, for example, evolutionary constraints, mutation rates, types of mutations and their frequencies (e.g., single nucleotide polymorphism, larger genomic rearrangements) across species. The reconstruction of evolutionary changes over time using approaches from resurrection ecology in combination with (meta)genomic analysis and data on the biotic and abiotic changes could help us identify disadvantageous conditions and how viruses have adapted to them (Table 1). The isolation and comparison of viruses from different local populations in terms of their genetic differences, their life-history traits or their response to different conditions can further help in the classification of such conditions (Table 1). Experimental evolution studies, where evolutionary change is followed over time under controlled conditions, can be used to examine the evolvability of a virus species or strain and test it in response to defined conditions (Table 1), asking for example whether and how viruses evolve when they are exposed to strong UV radiation or hosts with disadvantageous C:N:P ratios. Overall, understanding the many conditions that lower viral success, the effects of such conditions and the potential of viruses to overcome them represents a fascinating research area within the growing field of algal virology that currently lacks understanding and should be addressed in future studies.

## Figures and Tables

**Table 1:** Overview of approaches to study viral adaptations to conditions that make them unsuccessful. We refer to viruses as unsuccessful when (i) there are low numbers of viruses in the environment; (ii) they have low or no viral infectivity and/or (iii) they have low or no viral replication ability and subsequent low or no release of viral particles into the environment. All approaches can be carried out at temporal and spatial scales.

Approach	Method	Examples *
**Comparative studies**		
	Sampling of communities over time and correlation with changes in extrinsic factors	[95]
	Resurrection ecology, correlation of abundances with changes in the environment	[119]
**Experimental studies**		
	Measurement of virus life cycle traits under different conditions	[83,85,100,101]
	Resurrection ecology, isolation of living viruses and measurement of life cycle traits under different conditions	
**Experimental evolution studies**		
	Virus evolution to different conditions (requires constant host)	
	Host-virus coevolution under different conditions	[87,120]
	Virus (co)evolution in communities under different conditions	[65]
**Modeling**		
	Virus population events across different conditions	[87]
**Genomics**		
	Viromics to check for absence/presence of viruses across different conditions	[18]
	Genomics and phylogenetic trees to decipher evolution and past population events (bottlenecks, extinctions, migrations, etc.)	[115,121,122]

* Examples of algal viruses are provided but approaches can be applied to all other viruses. No examples given indicates that we are not aware of studies using those approaches.
